# Prediction of hepatocellular carcinoma prognosis based on expression of an immune-related gene set

**DOI:** 10.18632/aging.102669

**Published:** 2020-01-12

**Authors:** Yuting He, Qin Dang, Jie Li, Qiyao Zhang, Xiao Yu, Miaomiao Xue, Wenzhi Guo

**Affiliations:** 1 Department of Hepatobiliary and Pancreatic Surgery, The First Affiliated Hospital of Zhengzhou University, Zhengzhou 450052, Henan, P.R. China; 2 Key Laboratory of Hepatobiliary and Pancreatic Surgery and Digestive Organ Transplantation of Henan Province, The First Affiliated Hospital of Zhengzhou University, Zhengzhou 450052, Henan, P.R. China; 3 Department of General Dentistry, The First Affiliated Hospital of Zhengzhou University, Zhengzhou 450052, Henan, P.R. China

**Keywords:** hepatocellular carcinoma, immune, gene expression, prognostic indicator

## Abstract

Hepatocellular carcinoma (HCC) is a common type of malignant tumor with an extremely poor prognosis. Because many HCC patients are diagnosed with advanced disease, surgical treatment is typically not possible, and other currently available treatments are often ineffective. Immunotherapy is being explored as a new treatment method for a variety of cancers, including HCC. However, there have been no systematic reports about the relationship between immune-related genes and HCC patient prognosis. In this study, we established and verified a gene set-based model to examine the relationship between immune-related genes and prognosis in HCC patients. The model was based on a dataset from The Cancer Genome Atlas (TCGA), and its stability and reliability was confirmed in four verification datasets. In addition, we performed multivariate Cox regression analyses to identify the independent risk factors affecting HCC patient prognoses. We found that this new model based on immune-related genes was effective for predicting prognosis, evaluating disease state, and identifying treatment options for HCC patients.

## INTRODUCTION

HCC, one of the most prevalent and life-threatening malignancies in the world, progresses rapidly and is difficult to treat. Most HCC patients are diagnosed with late-stage disease and present with distant metastases, portal vein tumor thrombus, and other morbid conditions, resulting in extremely poor prognoses [1–3]. Although chemotherapy, radiotherapy, and other treatment methods have modestly improved HCC patient survival rates in recent years, therapeutic outcomes are still largely unsatisfactory [4–6]. New treatment methods for advanced HCC are therefore needed to improve overall survival rates.

Immunotherapy has emerged as a promising potential treatment for a variety of cancers, including HCC [7, 8]. Previous research revealed that reactivation of NK cells and their cytotoxic activity against tumor cells can enhance anti-HCC effects [9]. In addition, immune-stimulating cytokines such as IFNG γ can inhibit HCC progression by inducing apoptosis or autophagy of HCC cells. However, due to inherent cancer variability, outcomes after immunotherapy are often unsatisfactory [10–13]. Increasing evidence indicates that expression of immune-related genes can be associated with tumor prognosis, and prognostic signatures based on these genes might help identify effective treatments for HCC patients [14]. The relationship between immune-related genes and prognosis therefore deserves further investigation.

The immune system plays an important role in the development and progression of HCC. The liver’s immunosuppressive microenvironment allows it to tolerate numerous exogenous intestinal bacteria and antigens that arrive via the portal vein. However, the liver is also unable to attack malignant tumor cells as a consequence. Combined immunotherapies are therefore often necessary to alter this pro-tumor microenvironment. Studies have shown that inhibiting PD-1 promotes vascular normalization and anti-tumor immune response in HCC [10, 15]. However, these studies involved small numbers of HCC patients, and the relationship between HCC immunotherapy and patient prognosis requires further investigation. A systematic characterization and analysis of the tumor immune microenvironment and its impact on prognosis is needed.

In this study, we integrated a multi-gene expression cohort of 903 cases to establish and validate an individualized immune-related gene set based on HCC prognostic signatures. Four independent datasets were also evaluated to verify the stability and reliability of our models. In addition, we performed a comprehensive analysis that incorporated clinical characteristic information to improve the accuracy of overall survival rate predictions.

## RESULTS

### Defining the single-sample immune gene set enrichment analysis

**Figure 1:**
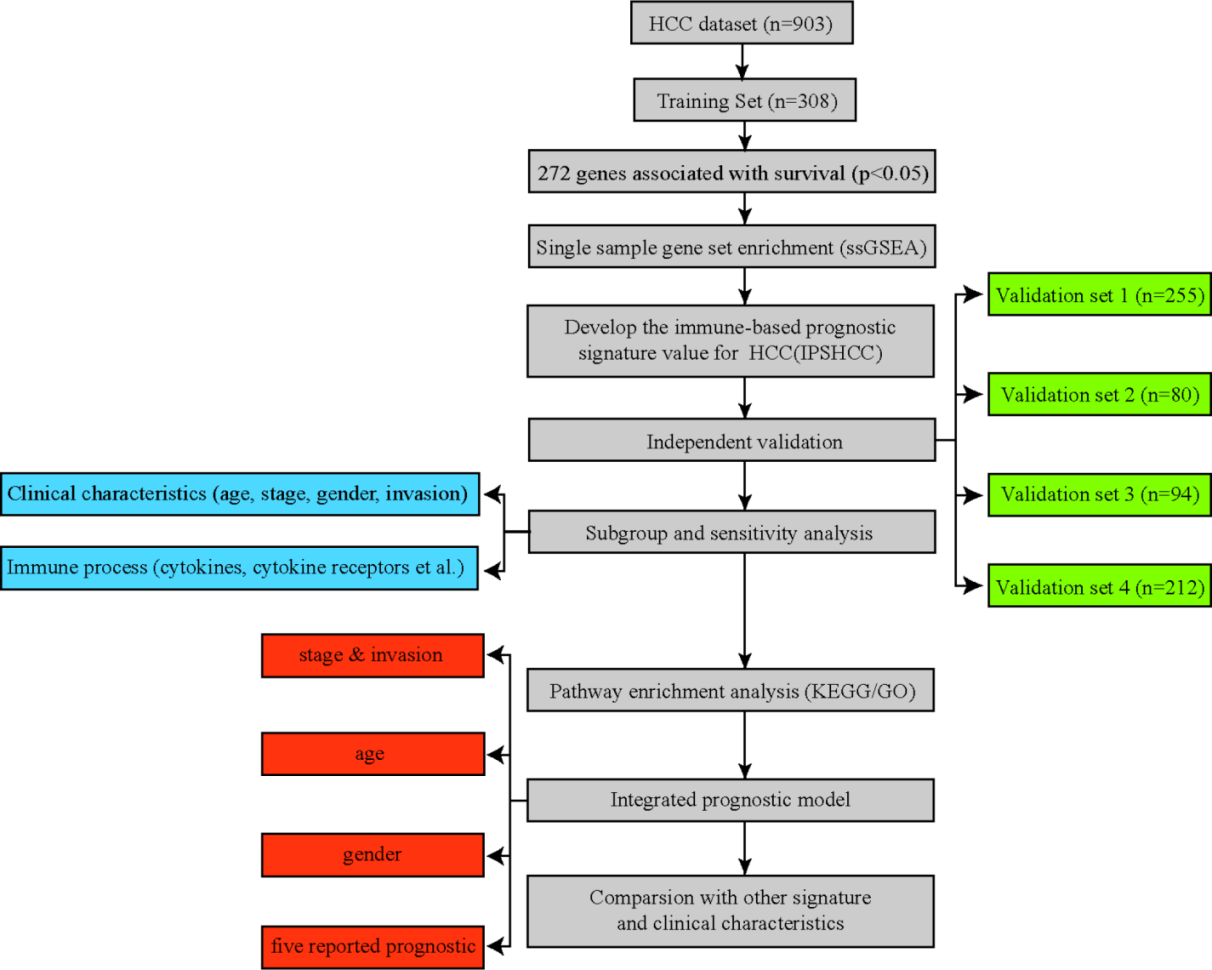
**Flowchart of the study. A total of 903 HCC patients from five separate datasets were included in the analysis.** We developed the immune-based prognostic signature for HCC (IPSHCC) using the training dataset and validated it in five independent validation subsets. We also integrated IPSHCC with stage, invasion, age, and gender to improve its prognostic value.

In total, 903 HCC patients from five datasets were included in the immune-based prognostic signature HCC (IPSHCC) analysis (Supplementary Table 1). In the training set, 1,810 genes representing 17 immune categories were identified; 196 of these genes representing 15 immune categories were related to overall survival. Those 196 genes were used as probes in single-sample gene set enrichment analysis (ssGSEA) of HCC patients to determine enrichment scores for each immune category (Figure 1). IPSHCC was defined as the comprehensive influence of coefficients generated by a multivariate Cox regression model on scores of different categories (Table 1). The median score of the training set patients (-0.0087) served as a cutoff value for dividing patients among low and high immunity risk groups in all datasets.

**Table 1:** Coefficients and multivariable Cox model results for each immune category in IPSHCC.

**Immune process**	**coef**	**HR**	**se(coef)**	**z**	**p**
Antigen Processing and Presentation	1.850399	6.362359	1.208288	1.531	0.12567
Antimicrobials	0.331736	1.393384	0.882671	0.376	0.70704
BCR Signaling Pathway	0.564767	1.759037	0.74325	0.76	0.44734
Chemokines	-1.060522	0.346275	0.546001	-1.942	0.0521
Chemokine Receptors	0.066627	1.068896	0.735582	0.091	0.92783
Cytokines	0.447841	1.56493	1.102603	0.406	0.68462
Cytokine Receptors	-2.791344	0.061339	0.913906	-3.054	0.00226
Interleukins	0.036797	1.037483	0.448511	0.082	0.93461
Interleukins Receptor	-0.242751	0.784467	0.572702	-0.424	0.67166
Natural Killer Cell Cytotoxicity	-3.251205	0.038728	1.31805	-2.467	0.01364
TCR signaling Pathway	0.171893	1.187551	1.505667	0.114	0.90911
TGFb Family Member	-0.772064	0.462058	0.547799	-1.409	0.15872
TGFb Family Member Receptor	-0.040184	0.960613	0.322612	-0.125	0.90087
TNF Family Members	-0.024008	0.976278	0.354878	-0.068	0.94606

### Verification of IPSHCC

**Figure 2:**
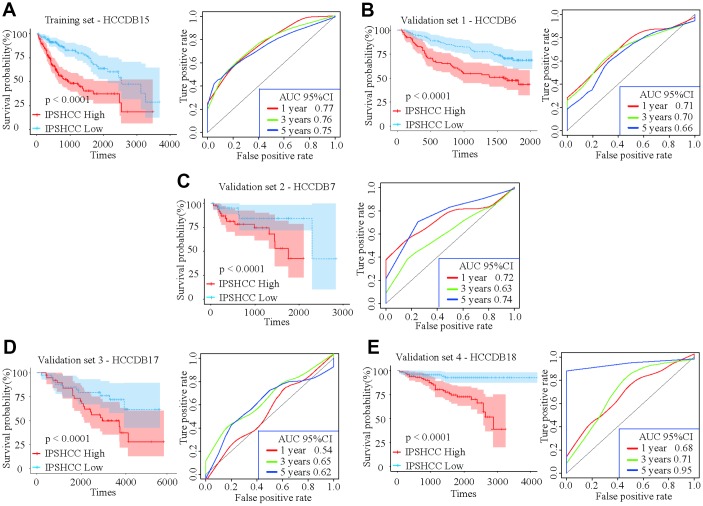
**Kaplan-Meier survival analyses of IPSHCC.** Patients in all five datasets were assigned to low-immune (blue) and high-immune (red) risk groups using median IPSHCC value as the cutoff. (**A**) In the training set, the survival probability of the “IPSHCC Low” group is higher than the “IPSHCC High” group (p < 0.0001). The 1, 3, and 5 year AUCs were 0.77, 0.76, and 0.75, respectively. (**B**–**E**) The IPSHCC prognostic signature was further validated in four independent validation sets. In each independent validation subset, survival probabilities were higher for the “IPSHCC Low” group than the “IPSHCC High” group (p < 0.0001). In validation sets 1 and 4, the 1-, 3-, and 5-year AUCs were > 0.65. In validation set 2, the 1-, 3-, and 5-year AUCs were 0.72, 0.63, and 0.74, respectively. In validation set 3, the 1-, 3-, and 5-year AUCs were ≤ 0.65.

**Figure 3:**
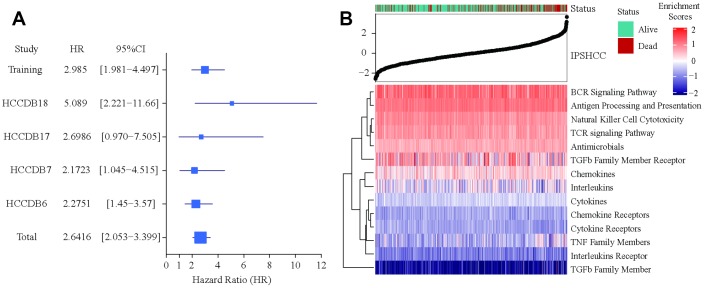
**Verification of IPSHCC.** (**A**) Meta-analysis of IPSHCC and overall survival in the five datasets. In the IPSHCC training set, the hazard ratio [HR] was 2.985 and the 95% confidence interval (CI) was 1.981-4.497 (p = 1.69 × 10^-6^). In the four verification sets, the HRs were between 2.1723 [95% CI: 1.045–4.515; p = 0.0377] and 5.089 [95% CI: 2.221–11.66; p = 0.0001]. In the integrated analysis of all datasets, the survival probability for the high immunity risk group was 2.6416 times lower than that of the low immunity risk group (HR = 2.6416; 95% CI: 2.053–3.399; p = 4.34 × 10^-14^). (**B**) IPSHCC distribution with survival status in the combined dataset. Upper half of panel: IPSOV distribution with patient survival status. The X axis is sorted by IPSHCC values. Red color indicates deceased patients, while green indicates living patients. Lower half of panel: Heatmap showing enrichment scores for the corresponding 15 immune categories.

Patients in the IPSHCC training set (hazard ratio [HR] = 2.985; 95% confidence interval (CI): 1.981–4.497; p = 1.69 × 10^-6^) and four verification sets (HR = 2.1723 [95% CI: 1.045–4.515; p = 0.0377] – 5.089 [95% CI: 2.221–11.66]) were divided between low and high immunity risk groups (Table 2). In the multivariate Cox model, even after controlling for age, stage, gender, and tumor invasion, immunity risk was still an independent prognostic factor (Figure 2A–2E). In an integrated analysis of all datasets, the probability of survival for the high immunity risk group was 2.6416 times lower than that of the low immunity risk group (HR = 2.6416; 95% CI: 2.053–3.399; p = 4.34 × 10^-14^) (Figure 3A). The distribution of IPSHCC with survival state in the composite dataset is shown in Figure 3B.

**Table 2:** IPSHCC values for high and low immunity risk patients in the training and verification datasets.

**Study**	**coef**	**HR**	**se(coef)**	**z**	**p**	**exp(coef)**	**exp(-coef)**	**lower.95**	**upper.95**	**n**
Training	1.0936	2.985	0.2091	5.231	1.69E-06	2.985	0.335	1.981	4.497	308
HCCDB18	1.627	5.089	0.423	3.847	0.00012	5.089	0.1965	2.221	11.66	212
HCCDB17	0.9927	2.6986	0.5218	1.902	0.0571	2.699	0.3706	0.9704	7.505	94
HCCDB7	0.7758	2.1723	0.3733	2.078	0.0377	2.172	0.4603	1.045	4.515	80
HCCDB6	0.822	2.2751	0.2299	3.575	0.00035	2.275	0.4395	1.45	3.57	209
Total	0.9714	2.6416	0.1287	7.55	4.34E-14	2.642	0.3786	2.053	3.399	903

### IPSHCC typing and sensitivity analysis

**Figure 4:**
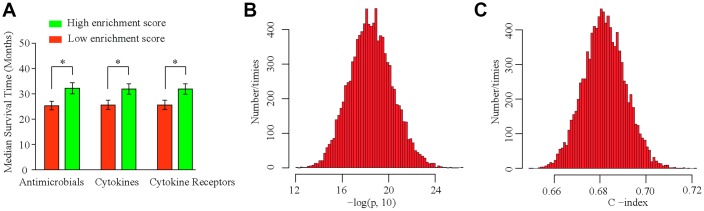
**IPSHCC typing and sensitivity analysis.** (**A**) Immune scores are calculated based on IPSHCC coefficients in antimicrobial, cytokine, and cytokine receptor immune processes. Scores were divided between low and high immune risk groups based on the median value. Median survival times were compared using the log-rank test. (**B**, **C**) To test the robustness of IPSHCC, we randomly re-sampled 500 cases from the consolidated datasets 10,000 times. (**B**) Histogram showing –log10 (P) values from the 10,000 resampled datasets. P-values were < 0.05 for all samples. (**C**) Histogram of C-index values from the 10,000 resampled datasets. The median C-index value was 0.6819, and standard deviation (SD) was 0.0091, demonstrating robust predictive ability.

We analyzed the sensitivity of our model depending on age, gender, stage, and tumor invasion to examine its stability in different clinical subgroups. IPSHCC was significant for all subgroups (Supplementary Figure 1), suggesting that it may be independent of clinical characteristics. In addition, we identified 196 genes associated with immune processes, including antimicrobials (38.27%), cytokines (26.53%), and cytokine receptors (25.51%) (Table 3). We calculated the immune score of the different subgroups for each immune process using the ssGSEA method. Patients with high immune scores had significantly longer median survival times for each process (Figure 4A). To test the robustness of IPSHCC, we randomly re-sampled 500 cases 10,000 times from the consolidated datasets. P-values of all samples were less than 0.05 in each re-sampling instance (Figure 4B and 4C). The median C-index value was 0.6819 and the standard deviation (SD) was 0.0091, demonstrating robust predictive ability.

**Table 3:** Genes (n = 196) participating in the immune process.

**Gene number**	**Ratio**	**Immune process**
75	38.27%	Antimicrobials
52	26.53%	Cytokines
50	25.51%	Cytokine_Receptors
22	11.22%	Antigen_Processing_and_Presentation
17	8.67%	NaturalKiller_Cell_Cytotoxicity
12	6.12%	Chemokine_Receptors
12	6.12%	TCRsignalingPathway
11	5.61%	Chemokines
7	3.57%	BCRSignalingPathway
4	2.04%	Interleukins
4	2.04%	Interleukins_Receptor
2	1.02%	TGFb_Family_Member
2	1.02%	TNF_Family_Members
2	1.02%	TNF_Family_Members_Receptors
1	0.51%	TGFb_Family_Member_Receptor

### Pathway enrichment analysis

Enrichment analysis for the 196 unique immune genes identified 69 related KEGG pathways (p < 0.05); for example, cytokines and cytokine receptors interacted with the MAPK, RAS, B/T-cell receptor, and PI3K/AKT signaling pathways. The PI3K/AKT signaling pathway regulates proliferation and survival of hepatoma cells, and abnormal activity in this pathway is associated with malignant transformation of hepatocytes, migration, adhesion, tumor angiogenesis, and degradation of the extracellular matrix. Tumor therapy strategies targeting the key molecules of the PI3K/AKT signaling pathway are currently being developed. In total, pathway analysis based on gene ontology identified 205 biological processes, 57 molecular functions, and 30 cellular constituent pathways representing a diverse spectrum of biological activities.

### Comparison with other prognostic signatures and clinical characteristics

**Figure 5:**
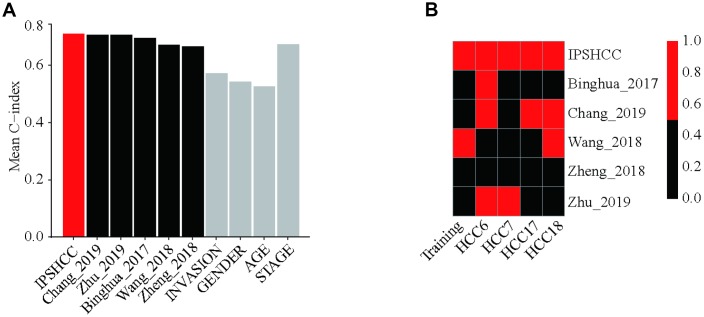
**Comparison with other prognostic signatures and clinical characteristics.** We calculated continuous prediction scores based on five other disease prognostic signatures and compared the different datasets using a univariate Cox model. (**A**) Mean C-index of IPSHCC, age, stage, gender, tumor invasion, and 5 reported signatures. Among 10 survival predictors, IPSHCC had the highest mean C-index (0.709) compared to age (0.526), stage (0.673), invasion (0.571), and gender (0.542). (**B**) P value comparison of IPSHCC and 5 reported signatures. Red block indicates the model is significant (P ≤ 0.05) while black indicates lack of significance (P > 0.05). IPSHCC demonstrated the lowest p-value among survival predictors (p = 9.22 × 10^-7^) across datasets.

After evaluating the accuracy and clinical consistency of IPSHCC modeling for predicting HCC, we calculated and compared continuous prediction scores according to five other disease prognostic signatures in different datasets using a univariate Cox model. Among 10 survival predictors, IPSHCC had the highest mean C-index (0.709) compared to age (0.526), stage (0.673), invasion (0.571), and gender (0.542) (Table 4, Figure 5A). The p-value of the IPSHCC prediction score was also the lowest among the survival predictors (p = 9.22 × 10^-7^) across datasets. (Table 4, Figure 5B).

**Table 4:** Continuous prediction score p-values from univariate Cox model.

**Signatures**	**p-value**	**C-index**
IPSHCC	9.22E-07	0.70962095
Chang_2019	0.51980352	0.70716302
Zhu_2019	0.48024456	0.70672321
Binghua_2017	0.08273801	0.6955979
Wang_2018	0.01635625	0.67220945
Zheng_2018	0.4165027	0.66521844
INVASION	0.29467416	0.57185805
GENDER	0.11775663	0.5420349
AGE	0.56301431	0.52690351
STAGE	6.80E-05	0.67329566

### Integrating IPSHCC and clinical characteristics

**Figure 6:**
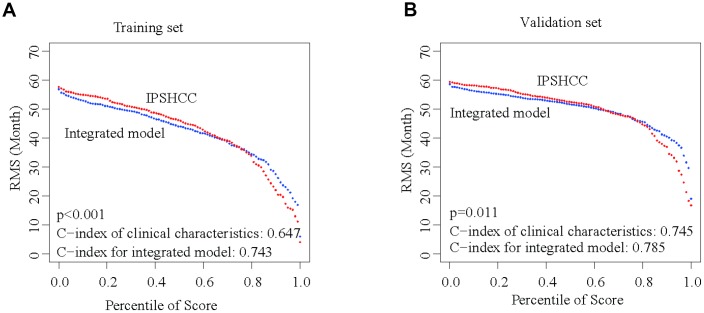
**Integration of IPSHCC and clinical characteristics.** To further enhance the predictive accuracy of IPSHCC, we integrated coefficients for clinical characteristics generated in the multivariate Cox regression model from the training and validation set. Restricted mean survival (RMS) curves for IPSHCC and the integrated model are shown. C-index values for the clinical characteristics alone and for the integrated model were compared. The p-value represents the difference between the C-index values for the two models. (**A**) In the training set, the C-index values of the clinical characteristics and the integrated model were 0.647 and 0.743, respectively (p < 0.001). (**B**) In the validation set, the C-index values of the clinical characteristics and the integrated model were 0.745 and 0.785, respectively (p = 0.011).

Besides IPSHCC, clinical characteristics such as age, gender, stage, and invasion were independent but complementary prognostic factors. To further enhance the predictive accuracy of IPSHCC, we integrated coefficients generated in the multivariate Cox regression model from the training set with IPSHCC (continuous score) as follows: (integrated model = 0.776924010 × IPSHCC + 0.004843653 × age + 0.625080315 × stage + 0.061769897 × gender - 0.319739104 × invasion). We then validated the integrated model using the HCCDB18 verification set, for which complete clinical information was available. The continuous score of the integrated model was only related to clinical characteristics (training set C-index: 0.743 vs. 0.647, p = 5.114 × 10^-5^; HCCDB18 C-index: 0.785 vs. 0.745, p = 0.011) and significantly improved the survival prediction (Figure 6A and 6B).

## DISCUSSION

Hepatocellular carcinoma (HCC) is the fourth leading cause (about 8.2%) of cancer-related death among men and women globally [16]. Despite the tremendous progress made recently in therapeutic strategies for HCC, outcomes for patients with advanced disease remain poor [2]. Identification of novel mechanisms of HCC progression and of effective targets is therefore crucial for improving HCC prognosis.

Various immune cells in the tumor microenvironment affect HCC development and progression [17–20]. Changes to the tumor microenvironment are closely associated with alterations in the immune system, and immune checkpoint inhibitors might help counteract the immunosuppressive effects of the HCC microenvironment [21–24]. Immunotherapies such as PD-1 [25] and CAR-T cells [26] have already been used to treat advanced HCC, and additional immunotherapies with novel targets might prove even more effective.

For the first time, we systematically studied the relationship between immune-related genes and prognosis in HCC patients. We confirmed that our IPSHCC model, together with clinical characteristics such as age, stage, gender, and tumor invasion, are independent prognostic factors for HCC. The immune-related genes included in the IPSHCC have been well characterized and have improved clinical adjunctive therapy and understanding of staging and progression in HCC [27, 28]. Xu et al*.* found that gene signatures associated with prognosis and immune infiltration in the renal cell carcinoma microenvironment might aid in the identification of effective immunotherapies [29]. Additionally, Shen et al*.* developed a promising prognostic signature and a method for evaluating clinical immunotherapies based on an immune gene set in ovarian cancer [30]. IPSHCC might aid in the discovery of new targets for molecular immunotherapies similar to those that have already proven effective in previous basic research and clinical studies.

Because IPSHCC is based on large sets of sequencing results obtained using different platforms and representing many patients, it generated more reliable prognostic predictions than other clinical characteristics. This allowed the classification of patients into different subgroups that might benefit from different personalized treatments based on their immune classification. However, IPSHCC only describes biomarkers; the biological mechanisms by which these biomarkers affect the development and progression of HCC require further investigation. Incorporation of additional patient follow-up data would also help improve the accuracy of this model for prognostic prediction.

In summary, our results demonstrate that IPSHCC is a promising model for predicting prognosis in HCC patients based on immune gene sets. Furthermore, this model might help identify novel therapeutic targets for advanced HCC.

## MATERIALS AND METHODS

### Gene expression profiles and study objectives

HCC gene expression profiles were retrospectively collected from the following five datasets: one from the Cancer Genome Atlas-Liver Hepatocellular Carcinoma (TCGA-LIHC) cohort, three from Gene Expression Omnibus (GEO), and one from the Liver Cancer Institute (LIRI-JP cohort). All patients had undergone a primary surgical excision of the tumor, and all pertinent clinical information, such as follow-up time, survival state, and gene expression levels was available. Our main objectives of this study were to compare overall survival of HCC patients with different immune characteristics.

### Processing of gene expression data

Only GEO cohorts with ≥ 80 subjects were used. Because a large number of samples in the TCGA-LIHC cohort had been characterized by RNA-seq gene expression (n = 308), we specifically examined microarray data from the Affymetrix Human Genome U133A array (n = 209) as described previously [29, 31]. Finally, data from the different GEO and TCGA-LIHC platforms were grouped into five independent datasets (Supplementary Table 1). An Entrez ID was utilized to represent each gene. The independent verification phase was executed as described previously [30]. All gene expression probes were individually adjusted in each dataset. Gene expression levels were logarithmically transformed before adjusting the batch effect.

### Identification of immune-related genes

We constructed a predictive signal gene set for immune-related genes which was divided into 17 categories based on molecular function, such as antimicrobials, cytokines, interleukins, T-cell receptor signaling, B-cell receptor signaling, and TNF family receptors. The details of genes in each category have been reported previously [32].

### Establishment of Immune-Based Prognostic Signature HCC (IPSHCC)

The immune-based prognostic signature HCC (IPSHCC) model was established as follows. We utilized the TCGA-LIHC cohort as the training set to screen genes associated with overall survival. The immune-related gene set included 1,810 genes, of which 1,356 were detected in the TCGA-LIHC cohort. We utilized the Cox proportional hazards model to evaluate the effect of each gene in combination with age, stage, invasion, and gender on overall survival. Genes with p-values greater than 0.05 were excluded. Next, we adopted single-sample gene set enrichment analysis (ssGSEA) to define an enrichment score representing the absolute enrichment of a gene in each sample of a given dataset as described previously [33]. Standardized enrichment scores were calculated for each immune category, and ssGSEA was conducted using the GSVA package for R. Finally, we established the IPSHCC model by combining the effect of every immune category in the training set. Multivariate Cox regression analysis was used to determine the coefficient of each category. Model: $\text{IPSHCC}=\Sigma_{i=1}^{K}\beta_{i}S_{i}$, where *S_i_* is the ssGSEA score of *i*^th^ immune category.

### Verification of IPSHCC

For a unified cutoff value, we divided patients into high- and low-risk groups. Gene expression levels were standardized in each dataset (the average value is 0, SD is 1). IPSHCC prognostic scores obtained from the training dataset were further analyzed using four verification datasets. For the multivariate Cox regression, age, stage, gender, and tumor invasion were all covariates.

### Pathway enrichment analysis

To further understand the function of the genes in IPSHCC, pathway enrichment analysis was conducted based on KEGG and GO databases as described previously [34]. Biological processes, molecular functions, and cellular constituents were included in the analysis. Multiple comparisons of p-values were made using the false discovery rate method, and all analysis was conducted in the R package cluster analysis program.

### Comparison with existing prognostic signatures

We collected five public prognostic signatures for comparison, including three to nine genes, to explore the survival classifications and predictive ability of IPSHCC. Continuous prognostic scores were calculated for each signature. Differences in continuous score p-values and population Cointegration statistics (C-index) from the univariate Cox model were compared in the five data sets.

### Statistical analysis

All data are shown as mean ± standard deviation (SD). For survival analysis, the Cox proportional hazards model was used to evaluate the relationship between gene signature and overall survival. Kaplan-Meier survival curves were plotted for each subgroup and compared with the Log-Rank Test. The R packages survival and survrm2 were used to estimate C-index values and mean survival rate curve limits, and the R package prelim was used to compare C-index values.

## Supplementary Material

Supplementary Figure 1

Supplementary Table 1

## References

[1] Hartke J, Johnson M, Ghabril M. The diagnosis and treatment of hepatocellular carcinoma. Semin Diagn Pathol. 2017; 34:153–59. 10.1053/j.semdp.2016.12.011 28108047

[2] Bruix J, Reig M, Sherman M. Evidence-Based Diagnosis, Staging, and Treatment of Patients With Hepatocellular Carcinoma. Gastroenterology. 2016; 150:835–53. 10.1053/j.gastro.2015.12.041 26795574

[3] Zhai W, Lim TK, Zhang T, Phang ST, Tiang Z, Guan P, Ng MH, Lim JQ, Yao F, Li Z, Ng PY, Yan J, Goh BK, et al. The spatial organization of intra-tumour heterogeneity and evolutionary trajectories of metastases in hepatocellular carcinoma. Nat Commun. 2017; 8:4565. 10.1038/ncomms14565 28240289 PMC5333358

[4] Yang JD, Roberts LR. Epidemiology and management of hepatocellular carcinoma. Infect Dis Clin North Am. 2010; 24:899–919, viii. 10.1016/j.idc.2010.07.004 20937457 PMC3949429

[5] Vilgrain V, Pereira H, Assenat E, Guiu B, Ilonca AD, Pageaux GP, Sibert A, Bouattour M, Lebtahi R, Allaham W, Barraud H, Laurent V, Mathias E, et al, and SARAH Trial Group. Efficacy and safety of selective internal radiotherapy with yttrium-90 resin microspheres compared with sorafenib in locally advanced and inoperable hepatocellular carcinoma (SARAH): an open-label randomised controlled phase 3 trial. Lancet Oncol. 2017; 18:1624–36. 10.1016/S1470-2045(17)30683-6 29107679

[6] Galle PR, Tovoli F, Foerster F, Wörns MA, Cucchetti A, Bolondi L. The treatment of intermediate stage tumours beyond TACE: from surgery to systemic therapy. J Hepatol. 2017; 67:173–83. 10.1016/j.jhep.2017.03.007 28323121

[7] Khalil DN, Smith EL, Brentjens RJ, Wolchok JD. The future of cancer treatment: immunomodulation, CARs and combination immunotherapy. Nat Rev Clin Oncol. 2016; 13:273–90. 10.1038/nrclinonc.2016.25 26977780 PMC5551685

[8] Hou J, Zhang H, Sun B, Karin M. The immunobiology of hepatocellular carcinoma in humans and mice: basic concepts and therapeutic implications. J Hepatol. 2020; 72:167–182. 10.1016/j.jhep.2019.08.014 31449859

[9] Xu W, Liu K, Chen M, Sun JY, McCaughan GW, Lu XJ, Ji J. Immunotherapy for hepatocellular carcinoma: recent advances and future perspectives. Ther Adv Med Oncol. 2019; 11:1758835919862692. 10.1177/1758835919862692 31384311 PMC6651675

[10] Garris CS, Arlauckas SP, Kohler RH, Trefny MP, Garren S, Piot C, Engblom C, Pfirschke C, Siwicki M, Gungabeesoon J, Freeman GJ, Warren SE, Ong S, et al. Successful anti-PD-1 cancer immunotherapy requires T cell-dendritic cell crosstalk involving the cytokines IFN-γ and IL-12. Immunity. 2018; 49:1148–1161.e7. 10.1016/j.immuni.2018.09.024 30552023 PMC6301092

[11] Garber K. Driving T-cell immunotherapy to solid tumors. Nat Biotechnol. 2018; 36:215–19. 10.1038/nbt.4090 29509745

[12] Sharma P, Allison JP. The future of immune checkpoint therapy. Science. 2015; 348:56–61. 10.1126/science.aaa8172 25838373

[13] Li J, Byrne KT, Yan F, Yamazoe T, Chen Z, Baslan T, Richman LP, Lin JH, Sun YH, Rech AJ, Balli D, Hay CA, Sela Y, et al. Tumor cell-intrinsic factors underlie heterogeneity of immune cell infiltration and response to immunotherapy. Immunity. 2018; 49:178–193.e7. 10.1016/j.immuni.2018.06.006 29958801 PMC6707727

[14] Gentles AJ, Newman AM, Liu CL, Bratman SV, Feng W, Kim D, Nair VS, Xu Y, Khuong A, Hoang CD, Diehn M, West RB, Plevritis SK, Alizadeh AA. The prognostic landscape of genes and infiltrating immune cells across human cancers. Nat Med. 2015; 21:938–45. 10.1038/nm.3909 26193342 PMC4852857

[15] Sharpe AH, Pauken KE. The diverse functions of the PD1 inhibitory pathway. Nat Rev Immunol. 2018; 18:153–67. 10.1038/nri.2017.108 28990585

[16] Bray F, Ferlay J, Soerjomataram I, Siegel RL, Torre LA, Jemal A. Global cancer statistics 2018: GLOBOCAN estimates of incidence and mortality worldwide for 36 cancers in 185 countries. CA Cancer J Clin. 2018; 68:394–424. 10.3322/caac.21492 30207593

[17] Kurebayashi Y, Ojima H, Tsujikawa H, Kubota N, Maehara J, Abe Y, Kitago M, Shinoda M, Kitagawa Y, Sakamoto M. Landscape of immune microenvironment in hepatocellular carcinoma and its additional impact on histological and molecular classification. Hepatology. 2018; 68:1025–41. 10.1002/hep.29904 29603348

[18] Gabrielson A, Wu Y, Wang H, Jiang J, Kallakury B, Gatalica Z, Reddy S, Kleiner D, Fishbein T, Johnson L, Island E, Satoskar R, Banovac F, et al. Intratumoral CD3 and CD8 T-cell densities associated with relapse-free survival in HCC. Cancer Immunol Res. 2016; 4:419–30. 10.1158/2326-6066.CIR-15-0110 26968206 PMC5303359

[19] Zhou SL, Zhou ZJ, Hu ZQ, Huang XW, Wang Z, Chen EB, Fan J, Cao Y, Dai Z, Zhou J. Tumor-Associated Neutrophils Recruit Macrophages and T-Regulatory Cells to Promote Progression of Hepatocellular Carcinoma and Resistance to Sorafenib. Gastroenterology. 2016; 150:1646–1658.e17. 10.1053/j.gastro.2016.02.040 26924089

[20] Dyck L, Lynch L. New Job for NK Cells: Architects of the Tumor Microenvironment. Immunity. 2018; 48:9–11. 10.1016/j.immuni.2018.01.001 29343443

[21] Dhanasekaran R, Nault JC, Roberts LR, Zucman-Rossi J. Genomic Medicine and Implications for Hepatocellular Carcinoma Prevention and Therapy. Gastroenterology. 2019; 156:492–509. 10.1053/j.gastro.2018.11.001 30404026 PMC6340723

[22] Zhu Y, Yang J, Xu D, Gao XM, Zhang Z, Hsu JL, Li CW, Lim SO, Sheng YY, Zhang Y, Li JH, Luo Q, Zheng Y, et al. Disruption of tumour-associated macrophage trafficking by the osteopontin-induced colony-stimulating factor-1 signalling sensitises hepatocellular carcinoma to anti-PD-L1 blockade. Gut. 2019; 68:1653–66. 10.1136/gutjnl-2019-318419 30902885

[23] Prieto J, Melero I, Sangro B. Immunological landscape and immunotherapy of hepatocellular carcinoma. Nat Rev Gastroenterol Hepatol. 2015; 12:681–700. 10.1038/nrgastro.2015.173 26484443

[24] Böttcher JP, Bonavita E, Chakravarty P, Blees H, Cabeza-Cabrerizo M, Sammicheli S, Rogers NC, Sahai E, Zelenay S, Reis e Sousa C. NK cells stimulate recruitment of cDC1 into the tumor microenvironment promoting cancer immune control. Cell. 2018; 172:1022–1037.e14. 10.1016/j.cell.2018.01.004 29429633 PMC5847168

[25] Shigeta K, Datta M, Hato T, Kitahara S, Chen IX, Matsui A, Kikuchi H, Mamessier E, Aoki S, Ramjiawan RR, Ochiai H, Bardeesy N, Huang P, et al. Dual PD-1 and VEGFR-2 blockade promotes vascular normalization and enhances anti-tumor immune responses in HCC. Hepatology. 2019. [Epub ahead of print]. 10.1002/hep.30889 31378984 PMC7000304

[26] Jiang Z, Jiang X, Chen S, Lai Y, Wei X, Li B, Lin S, Wang S, Wu Q, Liang Q, Liu Q, Peng M, Yu F, et al. Anti-GPC3-CAR T cells suppress the growth of tumor cells in patient-derived xenografts of hepatocellular carcinoma. Front Immunol. 2017; 7:690. 10.3389/fimmu.2016.00690 28123387 PMC5225101

[27] Greten TF, Sangro B. Targets for immunotherapy of liver cancer. J Hepatol. 2018; 68:157–166. 10.1016/j.jhep.2017.09.007 28923358 PMC5857416

[28] Greten TF, Wang XW, Korangy F. Current concepts of immune based treatments for patients with HCC: from basic science to novel treatment approaches. Gut. 2015; 64:842–48. 10.1136/gutjnl-2014-307990 25666193 PMC6311419

[29] Xu WH, Xu Y, Wang J, Wan FN, Wang HK, Cao DL, Shi GH, Qu YY, Zhang HL, Ye DW. Prognostic value and immune infiltration of novel signatures in clear cell renal cell carcinoma microenvironment. Aging (Albany NY). 2019; 11:6999–7020. 10.18632/aging.102233 31493764 PMC6756904

[30] Shen S, Wang G, Zhang R, Zhao Y, Yu H, Wei Y, Chen F. Development and validation of an immune gene-set based Prognostic signature in ovarian cancer. EBioMedicine. 2019; 40:318–26. 10.1016/j.ebiom.2018.12.054 30594555 PMC6412087

[31] He Y, Xue C, Yu Y, Chen J, Chen X, Ren F, Ren Z, Cui G, Sun R. CD44 is overexpressed and correlated with tumor progression in gallbladder cancer. Cancer Manag Res. 2018; 10:3857–65. 10.2147/CMAR.S175681 30288117 PMC6161708

[32] Bhattacharya S, Andorf S, Gomes L, Dunn P, Schaefer H, Pontius J, Berger P, Desborough V, Smith T, Campbell J, Thomson E, Monteiro R, Guimaraes P, et al. ImmPort: disseminating data to the public for the future of immunology. Immunol Res. 2014; 58:234–39. 10.1007/s12026-014-8516-1 24791905

[33] Tian X, Zhu X, Yan T, Yu C, Shen C, Hu Y, Hong J, Chen H, Fang JY. Recurrence-associated gene signature optimizes recurrence-free survival prediction of colorectal cancer. Mol Oncol. 2017; 11:1544–60. 10.1002/1878-0261.12117 28796930 PMC5664005

[34] Xue C, He Y, Zhu W, Chen X, Yu Y, Hu Q, Chen J, Liu L, Ren F, Ren Z, Cui G, Sun R. Low expression of LACTB promotes tumor progression and predicts poor prognosis in hepatocellular carcinoma. Am J Transl Res. 2018; 10:4152–62. 30662658 PMC6325492

