# Supplementary material for: Prediction of hepatocellular carcinoma prognosis based on expression of an immune-related gene set
Source: Aging (Albany NY). 2020 Jan 12;12(1):965–77. doi: 10.18632/aging.102669 (PMC6977696; doi:10.18632/aging.102669)
Supplement: Supplementary Figure 1 [file aging-12-102669-s002..pdf]

Supplementary Figure

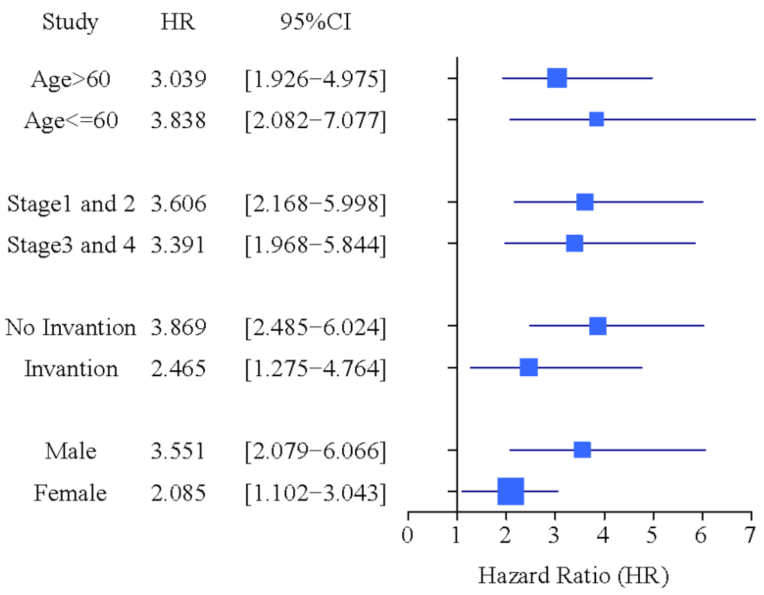

Supplementary Figure 1. Subgroup and sensitivity analysis for IPSHCC. We analyzed sensitivity according to age, gender, stage, and invasion to explore model stability for different clinical subgroups. IPSHCC was significant for all subgroups.
