# Supplementary material for: Prediction of hepatocellular carcinoma prognosis based on expression of an immune-related gene set
Source: Aging (Albany NY). 2020 Jan 12;12(1):965–77. doi: 10.18632/aging.102669 (PMC6977696; doi:10.18632/aging.102669)
Supplement: Supplementary Table 1 [file aging-12-102669-s001..pdf]

SUPPLEMENTARY TABLE

Supplementary Table 1. Summary of 903 HCC patients from 5 independent datasets included in the analysis.

Dataset ID	Source	Platform	No. of Adjacent	No. of HCC	Available No.	Drop reason
HCCDB6	GSE14520(GPL3721 Subset)	Affymetrix Human Genome U133A 2.0 Array	220	225	209	Missing survival information (n=16)
HCCDB7	GSE10143	Human 6k Transcriptionally Informative Gene Panel for DASL	82	80	80	-
HCCDB15	TCGA-LIHC	RNA-Seq	49	356	308	Missing survival information (n=48)
HCCDB17	GSE76427	Illumina HumanHT-12 V4.0 expression beadchip	52	115	94	Missing survival information (n=21)
HCCDB18	ICGC-LIRI-JP	RNA-Seq	177	212	212	-
**Totals**	**-**	**-**	**580**	**988**	**903**	**-**
